# Acute Lung Injury Caused by Mugwort Steaming

**DOI:** 10.7759/cureus.66229

**Published:** 2024-08-05

**Authors:** Hiroaki Ota, Kazunori Tobino

**Affiliations:** 1 Respiratory Medicine, Iizuka Hospital, Iizuka, JPN; 2 Respiratory Medicine, Iizuka Hospital, Fukuoka, JPN

**Keywords:** multidisciplinary discussion, bronchoalveolar lavage, occupational exposure, acute lung injury, mugwort steaming

## Abstract

Mugwort steaming is a traditional health practice with reported biological benefits, but its potential adverse effects on lung health remain unexplored. We report a case of a 48-year-old Japanese female who developed recurrent respiratory symptoms and abnormal lung shadows following occupational exposure to mugwort steaming. Initial diagnosis suggested nonfibrotic hypersensitivity pneumonitis. However, transbronchial lung cryobiopsy revealed findings consistent with acute lung injury (ALI). Multi-disciplinary discussion led to a final diagnosis of ALI caused by mugwort steaming. The patient's condition improved when mugwort steaming was discontinued. This case represents the first reported instance of ALI associated with mugwort steaming. It highlights the need for caution in traditional practices and emphasizes the importance of considering unconventional exposures in unexplained lung pathologies. Further research is warranted to establish the safety profile and potential risks of mugwort steaming.

## Introduction

Mugwort steaming is a type of traditional health practice in which mugwort is steamed and the steam is captured on the body surface. Several biological functions have been reported for mugwort, including anti-inflammatory, antimicrobial, insecticidal, antitumor, antioxidant, and immunomodulatory effects [1]. In recent years, there are facilities specializing in mugwort steaming, and it can also be done at home.

To the best of our knowledge, there have been no reports of acute lung injury caused by mugwort steaming. We herein report a case of acute lung injury (ALI) following mugwort steaming that was discussed in a multi-disciplinary discussion (MDD). The differential diagnosis of hypersensitivity pneumonitis was a major concern in this case. The patient's condition improved spontaneously during hospitalization, and she did not experience any recurrence after discharge as she refrained from further mugwort steaming.

## Case presentation

A 48-year-old Japanese female patient presented with recurrent sputum, cough, and abnormal lung shadows on computed tomography (CT). The patient had a history of follicular lymphoma, diagnosed two years prior, which was in remission following treatment in the hematology department. She was treated with six courses of bendamustine and five courses of obinutuzumab; abnormal lung shadows appeared on course five of obinutuzumab treatment, which persisted after discontinuation. The patient was interviewed in detail and it was found that mugwort steaming was started at the time when the abnormal shadows first appeared. Over the past year, she had been under follow-up with no particular symptoms, although abnormal lung shadows had repeatedly disappeared and recurred on chest radiographs and CT scans taken periodically to monitor for recurrence. Due to the persistence of these recurrent abnormal lung shadows, despite the absence of significant symptoms, she was referred to our department for further evaluation and management. She was not taking any regular medication or supplements. She had no smoking or drinking history. When we checked her professional history, we found that just before the abnormal lung shadows appeared, she had opened a mugwort steam shop and worked as a manager and instructor. She performed both face steaming and full-body steaming using mugwort. She has no allergies; her house is clean, and she has not bought any pets. Regarding pets, the patient did not have any pets of any kind, including birds, dogs, or cats. Nor were there any birds in her vicinity. She lived in a 42-year-old wooden building. She had used a humidifier for approximately 10 years. In the family history, her father had interstitial pneumonia, but we do not have detailed information about his condition. Some of her family members who had tried mugwort steaming did not report developing respiratory symptoms.

**Figure 1:**
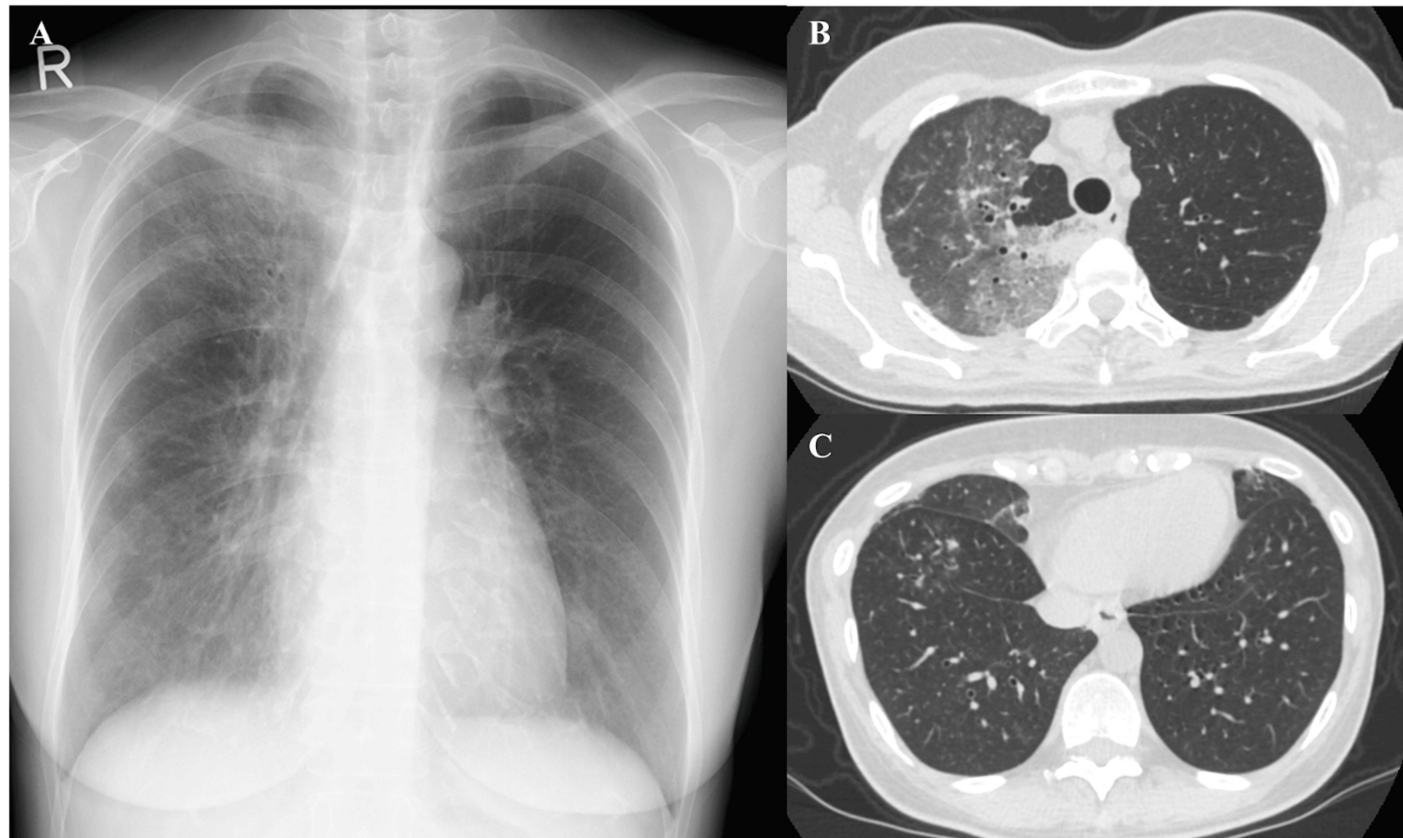
Chest imaging at initial presentation (A) Chest radiograph; B, C) Chest CT scans. Chest radiography and CT revealed ground-glass opacities predominantly in the right upper lobe and ill-defined centrilobular nodules distributed throughout both lungs

Her initial vital signs were as follows: oxygen saturation on room air, 99%; body temperature, 36.7°C; blood pressure, 130/88 mmHg; pulse rate, 120/min; respiratory rate, 16/min; level of consciousness, clear. On physical examination, heart sounds were normal, and the lungs were clear. No other specific findings were observed. Laboratory tests showed increased lactate dehydrogenase (LDH; 440 U/L), C-reactive protein (CRP; 2.6 mg/dL), KL-6 (636 U/mL), surfactant protein D (SP-D; 123 ng/mL), and negative results for antinuclear antibodies and collagen-related antibodies. Trichosporon asahii antigen blood test was negative (0.00). Chest radiography and CT revealed ground-glass opacities mainly in the right upper lobe, and ill-defined centrilobular nodules throughout the lung (Figure 1).

**Figure 2:**
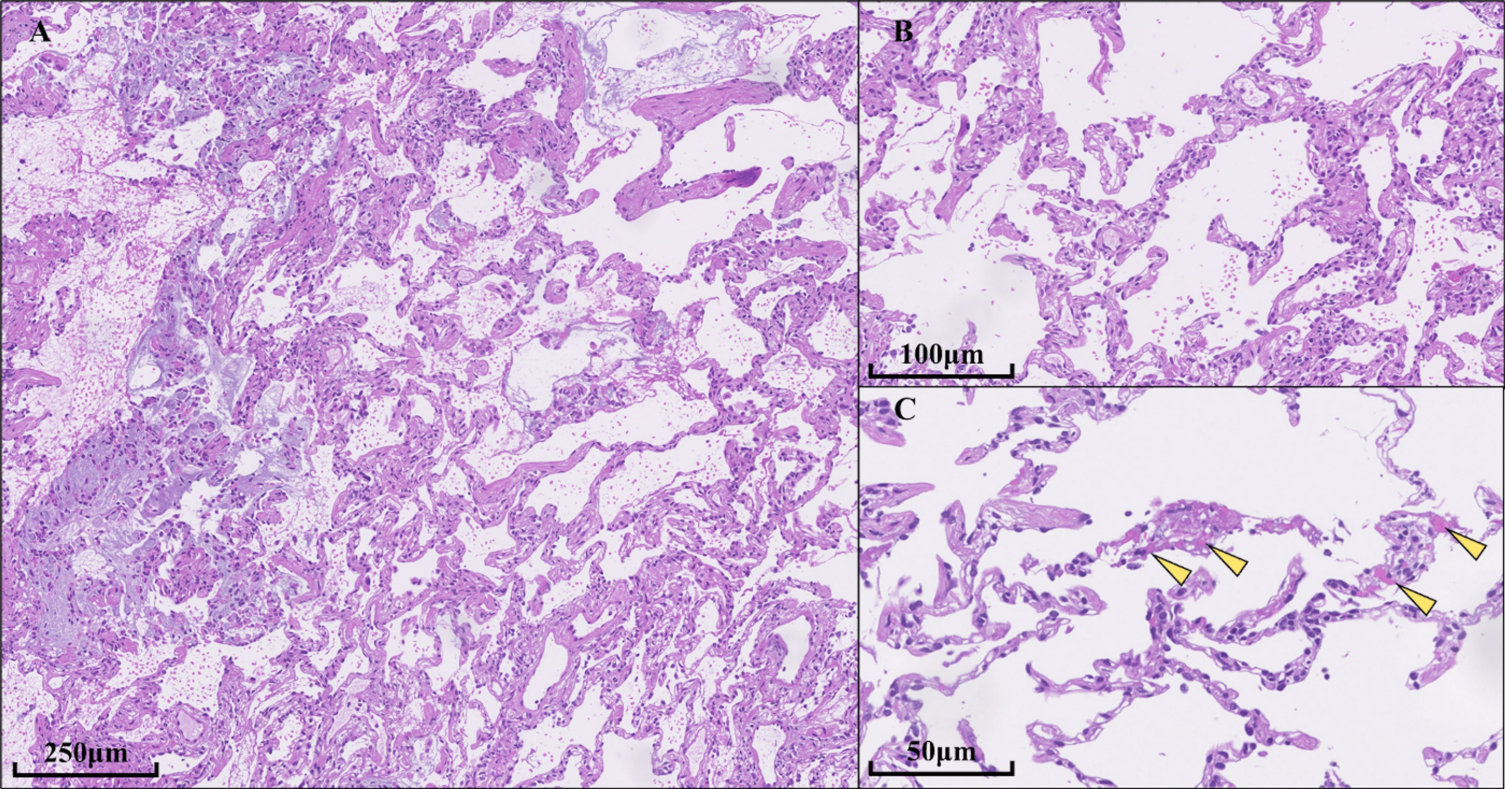
Histopathological findings from transbronchial lung cryobiopsy (TBLC) of the right lung. Hematoxylin and Eosin staining. A) low magnification; B, C) high magnification A, B) Histological analysis revealed diffuse lymphocytic infiltration of the alveolar septa, consistent with cellular non-specific interstitial pneumonia. C) No granulomas characteristic of hypersensitivity pneumonitis were observed. However, hyaline membranes and fibrin deposits were present, suggesting acute lung injury.

We performed a six-minute walk test and found no reduction in oxygenation. Bronchoalveolar lavage (BAL) revealed that the BAL fluid (BALF) was lymphocyte-dominant (55%). She was considered to have nonfibrotic hypersensitivity pneumonitis (NFHP) based on clinical and radiological diagnosis, her clear history of inhalation exposure to mugwort steaming, repeated abnormal lung shadows, CT findings consistent with an NFHP pattern as per guidelines, and lymphocyte dominance in the BALF [2]. Transbronchial lung cryobiopsy (TBLC) was performed from the right lung's B3a, B8a, and B9a regions, and histological analysis showed diffuse lymphocytic infiltration of the alveolar septa, consistent with cellular non-specific interstitial pneumonia (cNSIP). There were no granulomas characteristic of hypersensitivity pneumonitis, but hyaline membrane or fibrin deposits were found, suggesting acute lung injury (Figure 2).

**Figure 3:**
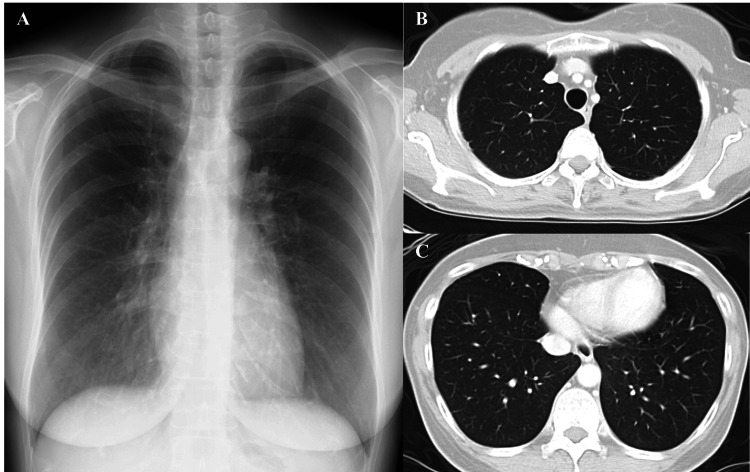
Chest imaging three months after discontinuation of mugwort steam therapy A) Chest radiograph. B, C) Chest CT scans. All previously observed abnormal opacities have resolved completely.

Based on these results, an MDD was performed, leading to a diagnosis of acute lung injury. As her symptoms were mild, she was instructed not to use mugwort face steaming and was followed up. Subsequently, there was an improvement in symptoms, blood tests, and imaging findings (Figure 3).

She continues to do full-body steaming without face steaming, but there has been no flare-up of her symptoms. This course of events also supported that it was an acute lung injury caused by mugwort steaming.

## Discussion

This is the first case report of acute lung injury caused by mugwort steaming. Occupational asthma caused by inhalation of mugwort was reported in 1996, suggesting that inhalation of the herb affects the airways [3]. However, since then, there have been no reports of acute lung injury due to mugwort steaming.

The diagnostic process was conducted through MDD in close consultation with radiologists and pathologists. The CT imaging pattern initially suggested non-fibrotic hypersensitivity pneumonitis (HP), but non-specific interstitial pneumonia (NSIP) was also considered. However, pathological examination showed no granulomas or multinucleated giant cells, ruling out hypersensitivity pneumonitis [2]. We carefully considered various differential diagnoses, including connective tissue disease-associated interstitial lung disease (CTD-ILD) and other forms of HP. However, there were no elevated antibodies suggestive of connective tissue disease and no physical findings suggestive of CTD. Additionally, the herbs used for mugwort steaming were pesticide-free, ruling out acute lung injury due to pesticides. The temporal relationship with the start of mugwort steaming suggests a strong causal relationship with this practice rather than with lymphoma treatment. We did not perform provocation tests, such as a home dust exposure test, due to the extensive ground-glass opacities present in both lung fields despite the patient being largely asymptomatic. We considered the potential risks of re-exposure to outweigh the benefits in this case. Since the patient showed improvement only by antigen avoidance, we consider this case to have a high degree of confidence in the diagnosis of acute lung injury caused by mugwort steaming. The clinical, radiological, and pathological findings in this case did not align with alternative diagnoses such as HP, NSIP, or CTD-ILD. Symptoms and shadows improved when the face was not steamed, and although the whole body is now steamed with reduced exposure to the face, the lack of recurrence of imaging findings suggests that exposure was also a factor.

Mugwort has long been reported to have multiple biological functions, such as anti-inflammatory, antibacterial, insecticidal, antitumor, antioxidant, and immunomodulatory effects [1]. Recently, antiviral effects against HBV and herpes viruses have also been reported [4]. Experiments with mice have shown that compounds extracted from mugwort inhibit cancer cells and induce apoptosis in vivo [5]. On the other hand, while mixing mugwort with chicken feed promotes antioxidant activity, it has been pointed out that high concentrations may disrupt the morphology of the intestinal tract and cause side effects [6]. The components of mugwort steam include chlorophyll, vitamins (A, B1, B2, and C), minerals (calcium and magnesium), essential oils (cineole and alpha-thujone), sesquiterpenes and organogermanium. Of these, alpha-thujone is reported to have the greatest potential health risk due to its neurotoxic effects at high doses. Essential oils can cause skin irritation and allergic reactions in some people. The safety of organic germanium is questionable, and some have been linked to kidney damage when taken orally in large quantities over a long period of time. However, the overall risk from mugwort steaming, when used as traditionally recommended, is considered low, as the concentration and absorption of these ingredients through ingestion or topical application and inhalation may differ. In addition, no reports of lung injury were found.

It is not known what concentrations and inhaled doses of mugwort steam are safe in the human lungs. Mugwort steaming can be performed in two main ways: facial steaming and full-body steaming. Facial steaming involves directing steam directly onto the face, which is then inhaled by the individual. This method concentrates the exposure primarily to the facial area and respiratory tract. In contrast, full-body steaming therapy requires the individual to wear a cloak or cover from the neck down, with steam applied to the body below the neck. This method potentially exposes a larger surface area of the body to the steam and its components. Importantly, facial steaming, compared to full-body steaming, leads to more direct inhalation of the steam, which could potentially increase the risk of lung injury. Interestingly, in this case, after the patient's symptoms improved, the patient switched to whole-body steaming, and the symptoms did not flare up. In mugwort steaming, powdered mugwort is placed in a pot, and the bottom is heated to generate steam. The generated steam particles typically range from 1-5 µm, which generally reach the peripheral airways, and the particles are expected to reach the lower respiratory tract and alveoli [7]. This particle size distribution may contribute to the potential for lung injury, particularly with facial steaming where inhalation is more direct.

## Conclusions

Although mugwort steaming has been practiced since ancient times, it is not yet a folk remedy with established efficacy and safety. This case report, presenting the first documented instance of acute lung injury caused by mugwort steaming, underscores the potential risks associated with this practice, particularly when involving direct inhalation of mugwort steam. This case highlights the significance of exposure methods, suggesting that face steaming may pose a higher risk to the respiratory system compared to whole-body steaming.

This case emphasizes the importance of thorough clinical history, including occupational exposures, in diagnosing unusual cases of lung injury. It also demonstrates the value of multidisciplinary discussions in reaching accurate diagnoses for complex cases. While mugwort has been reported to have various beneficial biological functions, this case raises concerns about its safety when used in steam form, especially at unknown concentrations. Further research is crucial to establish safe exposure levels and methods of use for mugwort steaming. Healthcare providers should be aware of this potential complication and educate patients about the possible risks, especially when performed frequently or with direct facial exposure. Given the increasing popularity of mugwort steaming, regulatory bodies may need to consider guidelines or safety standards for this practice, particularly in commercial settings.
